# Middle-preserving pancreatectomy: report of two cases and review of the literature

**DOI:** 10.1186/1477-7819-11-106

**Published:** 2013-05-23

**Authors:** Kun Cheng, Bai-yong Shen, Cheng-hong Peng, Li-ma Na, Dong-feng Cheng

**Affiliations:** 1 Department of General Surgery, Rui Jin Hospital, Shanghai Jiao Tong University School of Medicine, 197, Ruijin Er Road, Shanghai 200025, China; 2 Department of endocrinology, The First Teaching Hospital of Xinjiang Medical University, 1, Liyushan Road, Urumqi 830000, China

**Keywords:** Parenchyma-sparing, Pancreatectomy, Outcomes

## Abstract

**Background:**

Middle-preserving pancreatectomy (MPP) is a parenchyma-sparing surgical procedure which has recently been sporadically reported for the treatment of multicentric periampullary-pancreatic lesions. However, a comprehensive recognition of this procedure has not been clearly elucidated.

**Case presentation:**

We herein report two patients undergoing MPP due to synchronous multicentric pancreatic neoplasm. Patient one was a 24-year-old woman with a multicentric solid pseudopapillary neoplasm (SPN) and patient two was a 36-year-old woman with a multicentric serous cystic neoplasm (SCN). Simultaneous atypical pancreaticoduodenectomy and atypical left pancreatectomy were performed in patient one; simultaneous standard pancreaticoduodenectomy and atypical left pancreatectomy with spleen preservation were performed in patient two. Approximately 6 cm and 5 cm segments of the middle portion of the pancreas were preserved, respectively. At follow-up at 36 months and 6 months respectively, patient one had developed diabetes and malabsorption requiring dietary control, exercise and pancreatic enzyme supplement whereas patient two showed normal fasting blood glucose without diarrhea. Both patients were disease-free and in good nutritional condition. We reviewed twenty cases of MPP that were previously reported in the literature. Patient characteristics, surgical techniques and short- and long-term outcomes were analyzed.

**Conclusion:**

MPP is mainly beneficial for multicentric noninvasive periampullary-pancreatic lesions. However, for multicentric periampullary-pancreatic lesions involving even primary invasive cancers, as long as the invasive cancers affect only one side of the pancreas (proximal or distal), MPP could serve as a rational choice in well-selected patients.

## Background

In recent years, there has been an increasing interest in parenchyma-sparing pancreatic surgery for benign, borderline and low-grade malignant lesions involving isolated or multicentric portions of the pancreas, especially in young patients with long life expectancies. The aim is of preserving exocrine and endocrine pancreatic function, and achieving a better quality of life after operation [1-3].

Middle-preserving pancreatectomy (MPP) is a surgical procedure recently advocated as treatment for multicentric periampullary-pancreatic lesions in which the middle portion of the pancreas is unaffected; it serves as an alternative to total pancreatectomy (TP) with the goal of preventing ensuing endocrine or exocrine pancreatic insufficiency after TP without compromising oncological resection. MPP is composed of either two pancreatic surgical approaches simultaneously (one-stage) or as a two-stage method; one approach for lesions located in the pancreatic head and periampullary region - the pylorus-preserving pancreaticoduodenectomy (PPPD) for example, and the other - which is the atypical left pancreatectomy (LP) [1,4-13], is for lesions confined to the pancreatic body/tail region. However, due to the rarity of suitable cases, MPP is a very uncommon surgical procedure, resulting in difficulty in elucidating its place in pancreatic surgery.

In this present article, the authors presented their own experience of two patients undergoing simultaneous MPP. In addition, a review of the literature on MPP published in English is included, in this report, with the aim of presenting a comprehensive recognition of MPP by analyzing its indications, surgical techniques, postoperative complications and long-term functional and oncologic results.

## Case presentation

### Case one

A 24-year-old woman was admitted to our department for the evaluation and treatment of a pancreatic mass which was found incidentally in a health check-up four months previously. She had no associated symptoms and her past medical history was unremarkable.

**Figure 1:**
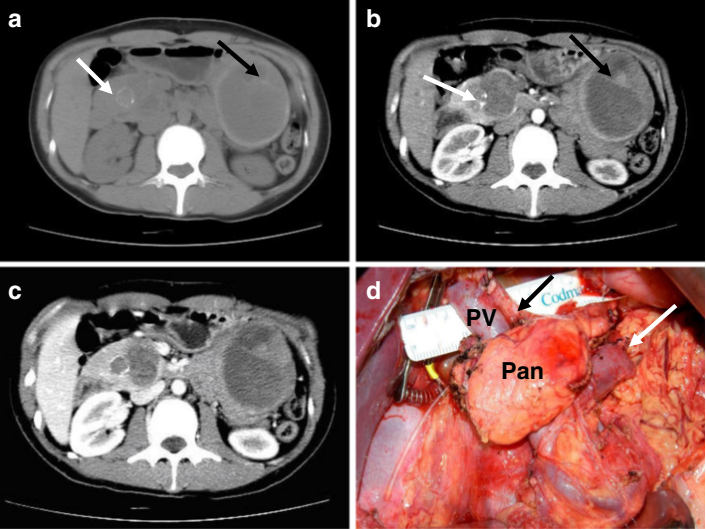
**CT scans and intraoperative imaging of patient one. a**) Plain CT revealed two well-defined heterogeneous lesions: calcification was shown in the pancreatic head tumor (white arrow) and a septum was shown in the tail tumor (black arrow). **b**) Enhanced CT showed peripheral enhancement and complex cystic components with areas of necrosis; calcification (white arrow) and septum (black arrow) were clearer (arrow). **c**) Enhanced CT scan in portal phase. **d**) After a simultaneous atypical PD and atypical LP, about 6 cm of the pancreatic body was preserved (Pan), proper hepatic artery (black arrow) and the stapled stump of the splenic vein (white arrow) can be seen. D, duodenum; G, gallbladder; Pan, Pancreas; T, tumor.

Physical examination was negative. Routine blood tests, liver function tests, pancreatic enzymes levels and serum tumor markers were all in the normal range. Body mass index was 20.3 kg/m^2^ (52 kg/1.6 m^2^). Abdominal computed tomography (CT) was performed, which showed two well-defined, low attenuation masses with peripheral enhancement and complex cystic components with areas of necrosis and calcification in the head and the tail of the pancreas (Figure 1a-c).

The patient was diagnosed with a multicentric solid pseudopapillary neoplasm (SPN). Due to the low malignant nature of SPN, a surgical procedure consisting of pancreaticoduodenectomy (PD) and atypical LP with splenectomy was planned. During operation, two tumors located in the head and the tail of the pancreas were identified; the distal pancreatic stump along with splenic vessels was stapled about 2 cm proximal to the pancreatic tail tumor. An approximate 6 cm segment of normal pancreatic tissue was preserved after a simultaneous atypical PD and atypical LP (that is, transection line not to portal vein/superior mesenteric vein) with splenectomy was performed (Figure 1d). Diagnosis of SPN for both tumors was made and the proximal and distal margins were demonstrated to be negative for tumor by intraoperative frozen section. For the proximal pancreatic stump, a retrocolic, two-layer, end-to-side invagination pancreaticojejunostomy (PJ) and a one-layer, end-to-side hepaticojejunostomy were performed, followed by a two-layer end-to-end antecolic gastrojejunostomy. Both of the tumors arising from two foci were diagnosed as SPN at final pathological examination. The estimated entire normal pancreatic volume was 36.6 cm^3^ and about 47.2% of normal parenchyma was preserved (20.2 cm^3^) through CT volumetric assessment.

The postoperative course was uneventful. Postoperative blood glucose levels ranged from 5.5 to 11.2 mmol/L and the fasting blood glucose was 5.1 mmol/L one month after operation. The patient developed diabetes and malabsorption 34 months after surgery, but her glycemic control was achieved by diet and exercise, and diarrhea was controlled by pancreatic enzyme supplement. To date, 36 months disease-free survival has been observed with no weight loss.

### Case two

**Figure 2:**
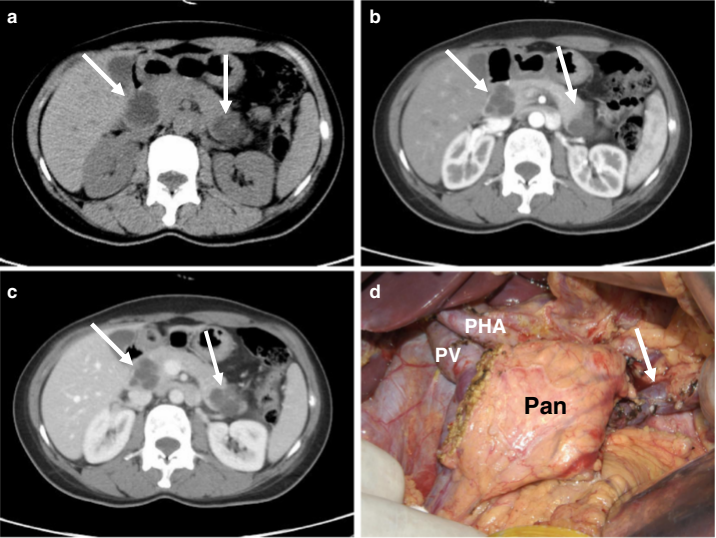
**CT scans and intraoperative imaging of patient two. a**) Plain CT revealed two well-defined cystic lesions (white arrow). **b**) Enhanced CT demonstrated polycystic appearance of the tumor with no enhancement in the arterial phase (white arrow). **c**) Enhanced CT demonstrated polycystic appearance of the tumor with no enhancement in portal phase (white arrow). **d**) After a simultaneous standard PD and spleen-preserving atypical LP, about 5 cm of the pancreatic body (Pan) and the splenic vein (arrow) were preserved. Pan, pancreas; PHA, proper hepatic artery; PV, portal vein.

A 36-year-old woman was found incidentally in a health check-up to have multicentric pancreatic cystic lesions measuring 23 mm and 24 mm in diameter. These were located synchronously in the head and the tail, respectively. She was asymptomatic and her past medical history was unremarkable. Multicentric serous cystic neoplasm (SCN) was diagnosed and the patient was followed-up with three-monthly US scans. During the follow-up, a slight, progressive increase in tumor diameter was apparent on subsequent US evaluation. After 27 months, the lesions were 31 mm and 40 mm in diameter respectively and the patient began to experience upper abdominal discomfort. Due to the increasing size of the lesions and her symptoms, she was then admitted to our department for treatment of the pancreatic mass. A further CT scan showed two well-defined cystic lesions located in the head and tail of the pancreas (Figure 2a-c). Her routine laboratory tests were within normal range, including serum tumor markers CEA and CA19-9. Body mass index was 20.1 kg/m^2^ (58 kg/1.7 m^2^) on admission.

Considering the natural biological behavior of SCN, a more conservative procedure was planned preoperatively: duodenum-preserving pancreatic head resection (DPPHR) for tumor located in the head and spleen-preserving atypical LP for tumor in the tail. On laparotomy, however, the lesion in the head was found to be strongly attached to the descending duodenum, making DPPHR impossible. Consequently, we performed a standard PD and spleen-preserving atypical LP for this patient. The proximal and distal margins were demonstrated to be negative for tumor by intraoperative frozen section. An approximate 5 cm segment of normal pancreatic tissue was preserved in this patient (Figure 2d). During the reconstruction phase, in contrast to patient one, end-to-side, duct-to-mucosa PJ was applied; however, the remaining reconstruction was the same as in patient one. Diagnosis of SCN for both tumors was confirmed at pathological examination. The estimated entire normal pancreas volume was 47.8 cm^3^ and about 50.8% of normal parenchyma was preserved (24.3 cm^3^) through CT volumetric assessment.

The patient developed delayed gastric emptying (DGE) which was managed successfully in a conservative way and six months disease-free survival without exocrine or endocrine pancreatic insufficiency has been observed so far. The patient is also in good condition, takes no digestive enzymes, has no diarrhea and her blood glucose was normal at the last examination with a fasting blood glucose is 5.3 mmol/L.

## Discussion

Recent trends in pancreatic surgery favor parenchyma-sparing over radical resection, where appropriate, for pancreatic lesions, and operations such as middle- pancreatectomy (MP) and enucleation, have gained satisfactory results in terms of achieving better functional preservation without compromising oncological radicality [2,3]. Traditionally, in dealing with multicentric pancreatic lesions, TP has been the choice of surgery. However, the need for this major and demanding operation should be carefully balanced against patient life expectancy under an apancreatic condition, together with short- and long-term outcomes of this procedure. Actually, a national review in the USA revealed that TP carried a 28% major complication and 8.5% overall mortality rate perioperatively [14]. Additionally, in long-term follow-up, the Mayo Clinic found that 28% of patients after TP developed target organ complications and chronic diarrhea, 79% experienced episodic hypoglycaemia and 41% experienced severe hypoglycaemia [15]. Weighing these facts, the rationality of applying TP to multicentric periampullary-pancreatic lesions should be revisited. While TP is mandatory for multicentric invasive malignant lesions, it seems to be too extensive for benign, borderline or low-grade malignant conditions, especially in young, otherwise healthy, patients. Recently, a new surgical procedure, named as middle-segment preserving pancreatectomy by Miura *et al*. [1] in 2007 and MPP by Partelli *et al*. [6] in 2009, was proposed as an alternative of TP in well-selected patients [1,4-13].

To date, to the best of our knowledge, only 22 patients, including ours, have undergone this procedure. From data shown in Table 1, it is apparent that MPP is mainly acceptable for muticentric periampullary-pancreatic benign, borderline, low-grade malignant lesions and pancreatic metastases from other tumors in which lymphadenectomy is not necessary. However, six patients were diagnosed with primary periampullary-pancreatic invasive cancer [1,4,11,12]. One major concern that might be raised is whether MPP guarantees tumor-free margins and sufficiently extensive lymph node dissection in these situations. According to the surgical principles of treating pancreatic cancer [16], as long as the multicentric invasive cancers do not affect both the proximal and the distal portions of the pancreas, synchronously or metachronously, MPP should be pursued whenever possible since in theory, it would offer sufficient extent of resection and nodal clearance, as demonstrated in previous reports [1,11,12]. Although a two-stage MPP was applied in one patient who presented with multicentric metachronous pancreatic adenocarcinoma [4], we would not advocate MPP in such situations because it evidently betrays the oncological surgical resection principle [16].

**Table 1:** Summary of reported cases of patients undergoing MPP

**First author/year**	**Type of MPP**	**Patient age/gender**		**Number of lesion**	**Location of lesions**	**Lesion pathology**	**Specific surgical intervention**^**d**^**/Reconstruction A, B, C,D**	**Preserved length of pancreas**	**Post operative morbidity**	**Post operative endocrine insufficiency**	**Post operative exocrine insufficiency**	**Follow up (months)**
Siassi/1999 [4]	two	62/F		2	body/tail	PDAC/PDAC	atypical PPPD/ atypical LP^eg^/B	5 cm	no	yes	yes	NED (12)
Lloyd/2003 [5]	one	31/F		2	head/body/tail	SPN/SPN	PPPD/ atypical LP^e^/D	2 cm	pseudocyst intervention	no	no	NED (17)
Miura/2007 [1]	two	66/M		2	head/tail	bIPMN (adenoma)/PDAC	PD ^g^/ atypical LP^e^/A	4 cm	no	no	no	NED (20)
	two	66/M		2	head/tail	Vater carcinoma/IPMN (adenoma)	PPPD/ atypical LP^fg^/A	5 cm	no	no	no	NED (10)
	one	70/M		2	head/tail	Vater carcinoma/ bIPMN (adenoma)	PPPD/ atypical LP^e^/A	6 cm	grade B PF	no^a^	no	NED (6)
Partelli/2009 [6]	one	28	3M/2F	3	head/tail	NF-PET /NF-PET	4PPPD,1PD/5 atypical LP^e^/A	NA	1 patient grade A PF	no	yes	NED (118)
	one	32		2	head/tail	NF-PET (carcinoma)/ NF-PET (carcinoma)		NA		no	no	NED (22)
	one	70		5	head/tail	bIPMN/bIPMN		NA		yes	yes	NED (20)
	one	35		2	head/tail	bIPMN/CP		NA		no	no	NED (18)
	one	60		2	head/tail	retention cyst/CP		NA		yes	yes	NED (14)
Chiang/2009 [7]	one	72/M		3	head/body/tail	mixIPMN (cancer *in situ* )/bIPMN /bIPMN (atypia)	extended PD/ atypical LP^e^/D	7 cm	no	no^a^	no	NED (36)
Kitasato/2010 [8]	one	65/F		4	head/body/tail	Metastatic RCC/Metastatic RCC	IPHR/ atypical LP^e^/D	40% volume	no	no	no	NED (31)
Ohzato/2010 [9]	one	67/F		5	head/body/tail	Metastatic RCC/ Metastatic RCC	atypical PPPD/atypical LP^e^/B	NA	bleeding reoperation	yes	no	NED (30)
Sperti/2010 [10]	one	59/M		2	head/tail	mixIPMN (borderline)/CP	PPPD/atypical LP^f^/A	5cm	bleeding intervention	yes	yes	NED (11)
Chen/2011 [11]	one	62/F		2	head/tail	Vater carcinoma/SPN	PD/ atypical LP^e^/D	NA	no	no	no	NED (6)
Horiguchi/2011 [12]	one	69/M		2	head/tail	bIPMN (adenoma)/bIPMN (adenoma)	IPHR/ atypical LP^e^/D	NA	grade B PF	no	no	Dead^h^ (16)
	one	67/F		5	head/tail	Gastrinoma/Gastrinoma	DPPHR/ atypical LP^f^/A	5 cm	grade B PF	no	no	NED (77)
	one	69/M		2	head/tail	bIPMN (adenoma)/bIPMN (adenoma)	IPHR/ atypical LP^f^/A	NA	grade B PF	yes^ac^	no	NED (14)
	one	83/F		2	head/tail	Bile duct cancer/bIPMN (adenoma)	SSPD/ atypical LP^f^/A	7 cm	no	no	no	NED (7)
Otani/2011 [13]	one	77/M		2	head/tail	bIPMN(adenoma)/mainIPMN(adenoma)	PPPD/ atypical LP^f^/C	6 cm	no	no^ab^	no^ab^	NED (84)
Ours	one	24/F		2	head/tail	SPN/SPN	atypical PD/ atypical LP^e^/A	6 cm	no	yes	yes	NED (36)
	one	36/F		2	head/tail	SCN/SCN	PD/ atypical LP^f^/B	5 cm	grade A DGE	no	no	NED (6)

CP, chronic pancreatitis; DGE, delayed gastric emptying; DPPHR, duodenum-preserving pancreatic head resection; IPHR, inferior pancreatic head resection; IPMN, intraductal papillary mucinous neoplasm; bIPMN, branch duct IPMN; mixIPMN, mix duct IPMN; mainIPMN, main duct IPMN; LP, left pancreatectomy; MPP, middle-preserving pancreatectomy; NA, not available; NED, no evidence of disease; NF-PET, nonfunctioning pancreatic endocrine tumor; one, one-stage MPP; PD, pancreaticoduodenectomy; PDAC, pancreatic duct adenocarcinoma; PPPD, pylorus-sparing pancreaticoduodenectomy; RCC, renal cell carcinoma; SCN, serous cystic neoplasm; SPN, solid pseudopapillary neoplasm; SSPD, substomach-preserving pancreaticoduodenectomy; two, two-stage MPP.

Reconstruction A, B, C, D: A, end-to-side pancreaticojejunostomy; B, end-to-side, duct-to-mucosa pancreaticojejunostomy; C, pancreaticogastrostomy; D, not available.

Postoperative endocrine insufficiency was defined as new-onset diabetes or preoperative insufficiency deteriorating. Postoperative exocrine insufficiency was defined as steatorrhea, weight loss requiring pancreatic enzymes supplementation, or preoperative insufficiency deteriorating. ^a^diabetes or exocrine insufficiency existed preoperatively; ^b^preoperative insufficiency improved after operation; ^c^preoperative insufficiency deteriorated after operation.

^d^(^e^withs splenectomy and ^f^without); ^g^in two-stage MPP, this operation is the previous one; ^h^died of malignant lymphoma.

No post-operative mortality occurred in any patient.

Despite multicentric noninvasive lesions being the perfect candidates for MPP, there has been an increasing number of reports where either tumor relapse or new primary lesions developed in the remnant pancreas after a previous pancreatectomy in IPMNs, PETs and metastatic RCC [17-19]. In order to avoid the recurrence in the remnant pancreas after MPP, appropriate candidates should be selected through careful preoperative evaluation of malignant potential, and intraoperative frozen section analysis of the two resection margins should be performed as a routine. Furthermore, intraoperative US would be helpful to identify all of the lesions and to confirm that the remnant was disease-free [6,7]. In a word, MPP should be converted to an oncologically appropriate operation if inadequate tumor resections are encountered intraoperatively.

Additionally, there might be another technical consideration when performing MPP. It has been reported that there is a relationship between the spleen and the endocrine pancreas on animal models [20,21]. Though the mechanism of their relationship is yet to be elucidated, splenocytes are considered to play an important role through accelerating β-cell neogenesis [20] and supporting endogenous β-cell recovery [21]. What is more, patients who underwent LP with splenectomy for chronic pancreatitis were reported to develop diabetes mellitus (DM) at a higher rate compared with those who underwent spleen-preserving LP [22,23]. Govil and Imrie [22] reported that splenic preservation was noted to reduce DM after LP to 15% compared with 56% after splenectomy at a median follow-up of 48 months; similarly, Hutchins *et al*. [23] concluded that DM developed in 43% patients with splenic preservation compared with 72% when the spleen was removed at median follow-up of 34 months. More recently, data from a research [24] has supported an association between trauma splenectomy and elevated mean blood glucose level. Interestingly, in current studies, new-onset DM and worsening DM were noted in 11.8% (2/7) of patients with splenic preservation, whereas 40% (6/15) of patients after splenectomy experienced new-onset DM. Surprisingly, patients in the splenic preservation group were older (median 67 years, range 36 to 83 years), which is thought to incur a higher risk of developing DM, compared with those in the splenectomy group (median 62 years, range 24 to 72 years). However, because of the intrinsic weaknesses of this study and the complexity of reported factors contributing to the development of DM after pancreatic resection [25], it is difficult to rationally interpret these data in relation to the role of splenic preservation. Nevertheless, it seems beneficial to preserve the spleen during MPP if oncologically and technically possible, which needs further observation.

Generally, MPP requires a pancreaticoenteric reconstruction where one stump drains the preserved pancreas leaving the other stump to be transected and closed. Thus, both that anastomosis and the transected and closed resection margin at the other stump are at risk for leakage, a situation similar to MP in which two sites with potential for pancreatic fistula formation exist [3]. A recent comprehensive review regarding MP found that rates of pancreatic fistula varied from 4% to 50%, morbidity from 13% to 62%, reoperation from 0 to 12%, and mortality from 0 to 3% [3]. In the current study concerning MPP, the overall morbidity was 40.9% (9/22), pancreatic fistula was 22.7% (5/22) and reoperation was 4.5% (1/22) with no mortality (Table 1), which is acceptable and comparable with the outcomes of MP.

The main benefit of MPP is that it spares the unaffected middle of the pancreas which might be sacrificed by TP. Early in 1973, DiMagno *et al*. [26] published a classic research which related the degree of malabsorption to the severity of pancreatic enzyme insufficiency. They showed that clinically significant malabsorption did not occur until 85 to 90% of pancreatic enzyme output was lost. With regard to the endocrine function, Slezak *et al*. [27] concluded that there was usually little change in glycemic control unless more than 80% of the pancreas was resected in patients with a previously normal pancreas. As reported by Yasuda *et al*. [28], the volume of the middle of the pancreas, as measured by CT-based pancreatic volumetry, was about 25% of the entire gland. So from theoretical point of view, MPP can preserve enough parenchyma to reduce the risk of developing endocrine and exocrine insufficiency in selected cases. In current study, at a median follow-up of 18 months (range 6 to 118), 8 out of 22 patients (36.4%) developed postoperative pancreatic exocrine and/or endocrine insufficiency (Table 1), which was obviously acceptable considering the apancreatic condition after TP. Furthermore, MPP preserves the glucagon-secreting alpha cells located in the pancreatic body, the loss of which is responsible for postoperative hypoglycemic episodes, which is still a difficult major problem after TP [15].

## Conclusions

Despite obvious drawbacks such as limited numbers and the heterogeneity of patients in the present article, it shows that MPP is mainly suitable for multicentric noninvasive periampullary-pancreatic lesions; for multicentric periampullary-pancreatic lesions even involving primary invasive cancers, MPP could serve as a rational choice in well-selected patients as long as the invasive cancers affect only one side of the pancreas.

## Consent

Written informed consent was obtained from the patients for publication of this case report and any accompanying images. A copy of the written consent is available for review by the editor-in-chief of this journal.

## Abbreviations

CP: Chronic pancreatitis; CT: Computer tomography; DGE: Delayed gastric emptying; DM: Diabetes mellitus; DPPHR: Duodenum-preserving pancreatic head resection; IPHR: Inferior pancreatic head resection; IPMN: Intraduct papillary mucinous neoplasm; LP: Left pancreatectomy; MPP: Middle-preserving pancreatectomy; NF-PET: Nonfunctioning pancreatic endocrine tumor; PD: Pancreaticoduodenectomy; PDAC: Pancreatic duct adenocarcinoma; PET: Pancreatic endocrine tumor; PHA: Proper hepatic artery; PV: Portal vein; RCC: Renal cell carcinoma; SCN: Serous cystic neoplasm; SPN: Solid pseudopapillary neoplasm; SSPD: Substomach-preserving pancreaticoduodenectomy; TP: Total pancreaticoduodenectomy.

## Competing interests

The authors declare that they have no conflict of interests.

## Authors’ contributions

Kun Cheng and Lima Na did the literature search and have been major contributors in writing the manuscript. Baiyong Shen revised the initial manuscript. Chenghong Peng did the final draft proof reading and approval. Dongfeng Cheng provided the images. All authors read and approved the final manuscript.
